# Relationship between the Necessary Support Level for Oral Hygiene and Performance of Physical, Daily Activity, and Cognitive Functions

**DOI:** 10.1155/2018/1542713

**Published:** 2018-11-04

**Authors:** Yoh Tamaki, Yoshimune Hiratsuka, Toshiro Kumakawa, Hiroko Miura

**Affiliations:** ^1^Department of Health and Welfare Services, National Institute of Public Health, 2-3-6 Minami, Wako, Saitama 351-0197, Japan; ^2^Department of Ophthalmology, Juntendo University School of Medicine, 3-1-3, Hongo, Bunkyo-ku, Tokyo 113-8431, Japan; ^3^The University of Fukuchiyama, 3370, Aza Hori, Fukuchiyama-shi, Kyoto 620-0886, Japan; ^4^Department of International Health and Collaboration, National Institute of Public Health, 2-3-6 Minami, Wako-shi, Saitama 351-0197, Japan

## Abstract

To maintain good oral hygiene on their own, elderly adults need comprehensive abilities, such as physical, daily activity, and cognitive functions. In the long-term care certification, care support specialists conduct surveys that include a total of 74 items about physical function, daily activity function, living functions, cognitive function, mental/behavioral disorders, and adaption to social life. The data of the long-term care certification survey contain three items related to oral health: the necessary support level for oral hygiene, ability to swallow, and assistance with food intake. The aims of this study were to identify which functions are absent in elderly individuals who cannot maintain proper oral hygiene and to clarify at which stage it is necessary to assist elderly individuals with their oral hygiene and provide professional oral care. In this study, an analysis was conducted to identify the relationship between the necessary support level for oral hygiene and the performance of physical, daily activity, and cognitive functions. The results of the long-term care certification surveys were analyzed for 23,423 cases that involved 9,571 individuals who submitted a claim using long-term care statements between January 2009 and March 2018. The results of a multivariable logistic regression analysis showed that the following items had high odds ratios: “walk” and “stand up” in the physical and daily activity functions and the ability to “understand the everyday routine” and “make daily decisions” in the cognitive functions. The results of a decision tree analysis revealed that in order for elderly individuals to maintain good oral hygiene on their own, they must have adequate physical functioning as well as adequate performance of cognitive functions. Our study's findings suggest that comprehensive ability in both physical and cognitive functions is required for elderly adults to maintain their oral hygiene.

## 1. Introduction

The population of elderly individuals in Japan has been dramatically increasing over the past three decades. According to a report by the Ministry of Internal Affairs and Communications of Japan, the percentage of older individuals aged 65 years or more increased from 7.1% in 1970 to 25.2% in 2013 [1]. One in every three individuals will be aged 65 years or older, and one in every five will be aged 75 years or older by 2035 [1]. By 2060, elderly adults will account for no less than 39.9% of the Japanese population, or 1 in 2.5 individuals, according to the medium-fertility projection [1].

Oral health is a significant factor affecting the quality of life and overall health and well-being of elderly individuals [2, 3]. In elderly adults, poor oral health affects the ability to chew and eat a variety of foods, causing poor dietary intake and weight loss [4, 5]. In addition, aspiration pneumonia is linked to poor oral health [6, 7]. Thus, maintaining good oral hygiene is important for elderly individuals [8, 9]. However, frail elderly individuals often have poor oral hygiene due to a decline in physical performance and cognitive function [10]. To maintain good oral hygiene on their own, elderly adults need comprehensive abilities, such as in their physical function, daily activity function, and cognitive function [11]. However, there is currently no research that has examined which functions are missing in elderly individuals who cannot maintain proper oral hygiene.

In Japan, long-term care insurance is a mandatory social insurance system, in which individuals who are aged 40 years or older are required to participate. Various long-term care services are provided for those who need care if they pay part of the cost [12]. Under the long-term care insurance, individuals aged 65 years or over (Category 1) and those aged 40–64 years (Category 2) are covered by the health insurance program. Long-term care insurance services are provided when individuals aged 65 years or over require care or support for any reason or when individuals aged 40–64 years develop age-related diseases, such as terminal cancer or rheumatoid arthritis, and thereby require care or support. The number of beneficiaries in Japan who required care under this system and eligible individuals (including those in need of support) was 2.18 million in 2000, which increased to 6.19 million in December 2015. Approximately 18% of Japanese people aged 65 years and older are eligible for long-term care certification [13].

To receive long-term care services in Japan, it is necessary to apply for the long-term care certification that is required in the municipality where each individual lives and has to be certified for the level of long-term care that they need. The survey for the need for long-term care certification assesses the mental and physical conditions of the certificate applicant and determines what level of care the applicant needs. This system is used to determine the necessary care level for several millions of individuals. The assessment data collected during this process are developed at the national level, are entered into a database, and are expected to be used for the analysis of regional and clinical issues, including quality of care. In the long-term care certification process, care support specialists conduct surveys that contain a total of 74 items about physical function, daily activity function, living functions, cognitive function, mental/behavioral disorders, and adaption to social life. The necessary care level is then evaluated using a total of seven levels (support need levels 1–2 and care need levels 1–5). The data of the long-term care certification survey contain three items related to oral health: the necessary support level for oral hygiene, ability to swallow, and assistance with food intake.

In this study, an analysis was conducted to identify the relationship between the necessary support level for oral hygiene and the performance of physical, daily activity, and cognitive functions. The aims of this study were to identify which functions are absent in elderly individuals who cannot maintain proper oral hygiene and to clarify at which stage it is necessary to assist elderly individuals with their oral hygiene and provide professional oral care.

## 2. Methods

The results of the long-term care certification surveys were analyzed for 23,423 cases that involved 9,571 individuals (3,640 males and 5,931 females; average age 85.8 ± 9 years, range 44–111 years) who submitted a claim using long-term care statements between January 2009 and March 2018. The study population was selected from 68,000 insured individuals in Mishima City, Shizuoka Prefecture (which has a total population of about 110,000). The results of the surveys conducted by the long-term care support specialists for the long-term care certification regarding physical function, daily activity function, living functions (10 items), cognitive function (eight items), mental/behavioral disorders, and adaption to social life were used. The data were analyzed to identify the relationship between the necessary support level for oral hygiene and the performance of other physical and daily activity functions (10 items: roll-over, get up, maintain a seated posture, maintain a standing position on both feet, walk, stand up, stand on one foot, vision, hearing, and swallowing) and cognitive function (eight items: communicate one's will, understand the everyday routine, state one's birthday and age, recall from short-term memory, state one's name, know what the current season is, know the environment, and make daily decisions).

First, cross-tabulation was performed of each item, including the necessary support level for oral hygiene and the evaluation items for the physical, daily activity, and cognitive functions. In addition, a multivariable logistic regression analysis was conducted with the necessary support level for oral hygiene as the dependent variable (total assistance—partial assistance/can do by their self) and the other performance evaluation items (a total of 20 items related to sex, age, physical function, daily activity function, and cognitive function) as the independent variables.

A more detailed analysis was conducted for similar items using a decision tree analysis; specifically, the Classification and Regression Trees method. The analyses were conducted using IBM SPSS version 24 and SPSS Modeler 17.1 (IBM Japan Ltd., Tokyo, Japan).

This survey was approved by the ethics committee of the National Institute of Public Health (NIPH-IBRA #12137) and the city council of Mishima City. All methods were performed in accordance with the International Ethical Guidelines for Epidemiological Studies [14], Guideline for the Provision of the Database for National Health Insurance Claim/Specific Medical Checkup/Specific Health Guidance [15], and Security Guidelines for Health Information Systems [16]. In compliance with the above guidelines, the researchers conducted the analyses once any information that could be identified as personal data was made anonymous by the local government.

## 3. Results

Table 1 shows the results of the cross-tabulation of the evaluation items of physical function and daily activity function, and the necessary support level for oral hygiene.  Table 2 shows the results of the cross-tabulation for the evaluation items of cognitive function and necessary support level for oral hygiene. The results of the multivariable logistic regression analysis showed that there was a significant odds ratio (OR) with regards to the necessary support level for oral hygiene for 16 items, including sex, and the evaluation items of the other physical, daily activity, and cognitive functions. Women had a lower risk in the necessary support level for oral hygiene than the men (OR 0.64). The following items had high ORs: walk (OR 2.3) and stand up (OR 2.10) in the physical and daily activity functions, and the ability to understand the everyday routine (OR 3.1) and make daily decisions (OR 2.55) in the cognitive function (Table 3). No significant OR was obtained for the ability to swallow, which is one of the ability indexes of oral health. For cognitive function, no significant OR was obtained in the ability to communicate one's will or state one's name.

**Figure 1:**
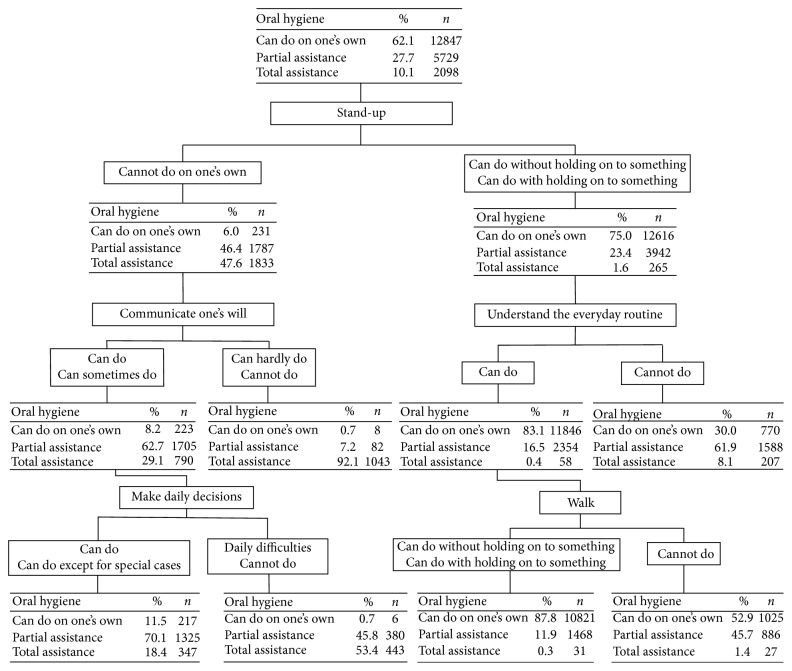
Results of decision tree analysis.

Furthermore, the results of the decision tree analysis revealed that 92.1% of respondents needed complete assistance with their oral hygiene if they could not stand on one's own and if they could not communicate their will (Figure 1). On the contrary, 83.1% of respondents did not require assistance with oral hygiene if they could stand or could stand if holding on to something, and could understand the everyday routine. In addition, more than 80% did not need assistance or partially needed assistance if they could or sometimes could communicate their will and if they could make daily decisions or could make daily decisions except in special cases, even if they could not stand up.

## 4. Discussion

This study found significant ORs for many of the survey items for physical function, daily activity function, and cognitive function regarding oral hygiene, which suggests that a comprehensive ability is required in terms of both physical function and cognitive function in order to maintain oral hygiene. Regarding the indexes of physical and daily activity functions, at least 80% of respondents were capable of maintaining oral hygiene with no assistance or partial assistance if they could or sometimes could not communicate their will, and if they could make daily decisions or could make daily decisions except for in special cases, even if they could not stand up. Therefore, for elderly individuals to maintain good oral hygiene on their own, they must have adequate physical functioning as well as adequate cognitive functioning.

Previous studies have confirmed that dementia can reduce the efficiency and stability of people to clean themselves; thus, the patients' oral hygiene condition worsens, and they develop more caries [17–19] and periodontal disease [20, 21] than people without dementia. Accordingly, it is presumed that patients with a moderate or higher level of dementia, who have many remaining teeth and who care for themselves without oral care assistance, have a high risk of oral diseases. For patients with mild dementia who can converse and understand well, there is a risk of multiple developments, such as periodontal diseases and caries, because oral self-care becomes poor owing to decreased self-motivation and finger dexterity, as well as visuospatial cognitive disorders. In this study, we found that respondents with a lower ability to understand everyday routines and to make daily decisions need assistance with oral hygiene because they are expected to have a higher risk of periodontal diseases and caries.

Physical functions deteriorate rapidly during dementia compared with during normal aging. It has also been reported that physiological vulnerability and vulnerability to stress increase the decline in general muscle mass and flexibility, as well as lowering the speed of a reaction to a fall, because physiological preparatory ability declines, and nutrition intake may decrease according to the level of severity [22, 23]. Moreover, the swallowing function deteriorates during Alzheimer's disease (AD), and there is a high risk of physical complications, such as aspiration pneumonia and acute disease episodes [24]. In the case of dementia with Lewy bodies, it is reported that decline in physical functions, such as swallowing functions, occur earlier than in AD [25]. Therefore, assistance with oral hygiene can be said to lead to a reduced risk of physical complications, such as aspiration pneumonia and the occurrence of acute diseases in elderly individuals with decreased cognitive function.

According to the report presented by the World Health Organization (WHO) entitled “Dementia: A Public Health Priority” (2012) [26], there are about 35.6 million individuals with dementia worldwide. The WHO estimates that this number will nearly double every 20 years, reaching an estimated 65.7 million in 2030 and 115.4 million in 2050 [27]. In 2013, a research group at the Ministry of Health, Labour, and Welfare reported that there are 4.62 million individuals with dementia in Japan [28].

If dementia is recognized in the early stages and early countermeasures are taken, patients will have more opportunities to receive support and become more independent. When plaque control suddenly deteriorates or when increased cavities and periodontal are noticed during a dental examination, it is important to treat elderly patients with the understanding that dementia is possible instead of assuming the condition is only the result of aging. In these patients, it is necessary to start a dental intervention, such as providing regular professional oral care, from the mild stage as much as possible, taking into consideration that dental treatment and special oral care become more difficult as dementia advances. In the dental treatment of patients with dementia, the proposed treatment plan should include a projection of the progress of the disease conditions as well as flexible measures that take into account the subsequent changes in condition. To achieve this, it is important to understand the dementia diseases and make appropriate plans for treatment and continuous care. Currently, patients with dementia who cannot visit dental clinics miss opportunities to receive dental treatment (including special oral care). These patients often do not request dental treatment until they develop bad breath, toothache, loosening teeth, difficulty with dentures, and difficulty eating. In many cases, by the time the family members or caregivers notice the change, the patient has already reached the stage where they cannot accurately describe the condition due to the severity of the dementia. In such cases, proactive dental treatment becomes difficult and, most of the time, only urgent care can be provided, which results in worse oral conditions. It is therefore important to understand the conditions of dementia and to continuously provide preventive dental treatment and oral health management for patients with dementia.

In this study, 23.2% of the elderly adults who required total assistance with oral hygiene had a swallowing disorder. On the contrary, 3% of those who had a swallowing disorder did not receive assistance with oral hygiene (data not shown). In the case of the care recipients who need total assistance, assistance with daily life is prioritized, and care often cannot be extended to oral hygiene. To better prevent aspiration pneumonia and other complications, providing assistance with oral hygiene at an early stage is crucial for elderly adults whose physical and cognitive functions have declined.

## 5. Conclusion

Our study's findings suggest that comprehensive ability in both physical and cognitive functions is required for elderly adults to maintain their oral hygiene. In the future, it will be necessary to assist elderly individuals with their oral hygiene and to provide professional oral care at an earlier stage for those who have reduced physical and cognitive functions.

## Figures and Tables

**Table 1:** Results of cross-tabulation for evaluation items of physical and daily activity functions and level of assistance needed for oral hygiene.

Ability items			Oral hygiene			Total	*p* value^*∗*^
			Can do on one's own	Partial assistance	Total assistance		
Roll-over	Can do without holding on to something	*N*	6963	1696	170	8829	<0.001
		%	78.9%	19.2%	1.9%	100.0%	
	Can do with holding on to something	*N*	5234	3131	397	8762	
		%	59.7%	35.7%	4.5%	100.0%	
	Cannot do on one's own	*N*	650	902	1531	3083	
		%	21.1%	29.3%	49.7%	100.0%	
Get up	Can do without holding on to something	*N*	1821	421	35	2277	<0.001
		%	80.0%	18.5%	1.5%	100.0%	
	Can do with holding on to something	*N*	10582	3556	249	14387	
		%	73.6%	24.7%	1.7%	100.0%	
	Cannot do on one's own	*N*	444	1752	1814	4010	
		%	11.1%	43.7%	45.2%	100.0%	
Maintain a seated posture	1: Can do on their own	*N*	7466	1823	130	9419	<0.001
		%	79.3%	19.4%	1.4%	100.0%	
	2: Can do if supported using one's own arms	*N*	3729	1673	130	5532	
		%	67.4%	30.2%	2.3%	100.0%	
	3: Can do with support	*N*	1631	2171	1617	5419	
		%	30.1%	40.1%	29.8%	100.0%	
	4: Cannot do	*N*	21	62	221	304	
		%	6.9%	20.4%	72.7%	100.0%	
Maintain a standing position on both feet	Can do without support	*N*	8447	1766	100	10313	<0.001
		%	81.9%	17.1%	1.0%	100.0%	
	Can do with support of something	*N*	4202	2627	297	7126	
		%	59.0%	36.9%	4.2%	100.0%	
	Cannot do on one's own	*N*	198	1336	1701	3235	
		%	6.1%	41.3%	52.6%	100.0%	
Walk	1: Can do without holding on to something	*N*	4622	850	63	5535	<0.001
		%	83.5%	15.4%	1.1%	100.0%	
	2: Can do with holding on to something	*N*	6975	2041	196	9212	
		%	75.7%	22.2%	2.1%	100.0%	
	3: Cannot do	*N*	1250	2838	1839	5927	
		%	21.1%	47.9%	31.0%	100.0%	
Stand up	Can do without holding on to something	*N*	1247	248	24	1519	<0.001
		%	82.1%	16.3%	1.6%	100.0%	
	Can do with holding on to something	*N*	11369	3694	241	15304	
		%	74.3%	24.1%	1.6%	100.0%	
	Cannot do	*N*	231	1787	1833	3851	
		%	6.0%	46.4%	47.6%	100.0%	
Stand on one foot	Can do without support	*N*	2435	400	14	2849	<0.001
		%	85.5%	14.0%	0.5%	100.0%	
	Can do with support of something	*N*	9445	2905	166	12516	
		%	75.5%	23.2%	1.3%	100.0%	
	Cannot do on one's own	*N*	967	2424	1918	5309	
		%	18.2%	45.7%	36.1%	100.0%	
Vision	Normal	*N*	11729	4862	880	17471	<0.001
		%	67.1%	27.8%	5.0%	100.0%	
	Cannot see the letters in newspapers or magazines but can see a picture one meter away	*N*	715	500	218	1433	
		%	49.9%	34.9%	15.2%	100.0%	
	Cannot see a picture one meter away but can see a picture directly in front of them	*N*	275	222	129	626	
		%	43.9%	35.5%	20.6%	100.0%	
	Cannot hardly see	*N*	119	88	50	257	
		%	46.3%	34.2%	19.5%	100.0%	
Hearing	Normal	*N*	9325	4006	1190	14521	<0.001
		%	64.2%	27.6%	8.2%	100.0%	
	Cannot hear a normal voice easily	*N*	2826	1169	361	4356	
		%	64.9%	26.8%	8.3%	100.0%	
	Can hear a loud voice	*N*	655	511	239	1405	
		%	46.6%	36.4%	17.0%	100.0%	
	Can hardly hear	*N*	40	42	19	101	
		%	39.6%	41.6%	18.8%	100.0%	
Swallowing	1: Can do on their own	*N*	12284	5188	1019	18491	<0.001
		%	66.4%	28.1%	5.5%	100.0%	
	2: Monitoring needed	*N*	547	513	593	1653	
		%	33.1%	31.0%	35.9%	100.0%	
	3: Cannot do	*N*	16	28	486	530	
		%	3.0%	5.3%	91.7%	100.0%	
Total		*N*	12847	5729	2098	20674	
		% of the total	62.1%	27.7%	10.1%	100.0%	

^*∗*^Chi-square test.

**Table 2:** Results of cross-tabulation for evaluation items of cognitive functions and level of assistance needed oral hygiene.

Item			Oral hygiene			Total	*p* value^*∗*^
			Can do on one's own	Partial assistance	Total assistance		
Communicate one's will	Can do	*N*	12272	4570	500	17342	<0.001
		%	70.8%	26.4%	2.9%	100.0%	
	Can sometimes do	*N*	526	926	457	1909	
		%	27.6%	48.5%	23.9%	100.0%	
	Can hardly do	*N*	47	224	696	967	
		%	4.9%	23.2%	72.0%	100.0%	
	Cannot do	*N*	2	9	445	456	
		%	0.4%	2.0%	97.6%	100.0%	<0.001
Understand the everyday routine	Can do	*N*	12042	3356	261	15659	
		%	76.9%	21.4%	1.7%	100.0%	
	Cannot do	*N*	805	2373	1837	5015	
		%	16.1%	47.3%	36.6%	100.0%	
State one's birthday and age	Can do	*N*	12588	4720	680	17988	<0.001
		%	70.0%	26.2%	3.8%	100.0%	
	Cannot do	*N*	259	1009	1418	2686	
		%	9.6%	37.6%	52.8%	100.0%	
Recall from short-term memory	Can do	*N*	10005	2618	255	12878	<0.001
		%	77.7%	20.3%	2.0%	100.0%	
	Cannot do	*N*	2842	3111	1843	7796	
		%	36.5%	39.9%	23.6%	100.0%	
State one's own name	Can do	*N*	12827	5605	1191	19623	<0.001
		%	65.4%	28.6%	6.1%	100.0%	
	Cannot do	*N*	20	124	907	1051	
		%	1.9%	11.8%	86.3%	100.0%	
Know what the current season is	Can do	*N*	11660	3436	389	15485	
		%	75.3%	22.2%	2.5%	100.0%	
	Cannot do	*N*	1187	2293	1709	5189	<0.001
		%	22.9%	44.2%	32.9%	100.0%	
Know the environment	Can do	*N*	12719	4966	751	18436	
		%	69.0%	26.9%	4.1%	100.0%	
	Cannot do	*N*	128	763	1347	2238	
		%	5.7%	34.1%	60.2%	100.0%	
Make daily decisions	Can do	*N*	7102	1099	79	8280	<0.001
		%	85.8%	13.3%	1.0%	100.0%	
	Can do except for special cases	*N*	5529	3409	361	9299	
		%	59.5%	36.7%	3.9%	100.0%	
	Daily difficulties	*N*	211	1159	953	2323	
		%	9.1%	49.9%	41.0%	100.0%	
	Cannot do	*N*	5	62	705	772	
		%	0.6%	8.0%	91.3%	100.0%	
Total		*N*	12847	5729	2098	20674	
		% of total	62.1%	27.7%	10.1%	100.0%	

^*∗*^Chi-square test.

**Table 3:** Results of multivariate logistic regression analysis.

	Item	Multivariate adjusted odds ratio	95% CI		*p* value
			Lower limit	Upper limit	
	Age	1.00	0.99	1.00	0.473
	Sex (female/male)	0.64	0.58	0.70	<0.001
Physical and daily activity functions	Roll-over	1.16	1.08	1.26	<0.001
	Get up	1.77	1.57	1.99	<0.001
	Maintain a seated posture	1.08	1.01	1.15	0.015
	Maintain a standing position on both feet	1.53	1.39	1.69	<0.001
	Walk	2.35	2.15	2.56	<0.001
	Stand up	2.10	1.81	2.43	<0.001
	Stand on one foot	1.31	1.18	1.46	<0.001
	Vision	1.19	1.10	1.29	<0.001
	Hearing	0.90	0.84	0.97	0.005
	Swallowing	1.15	0.99	1.34	0.077
Cognitive functions	Communicate one's will	1.12	0.97	1.28	0.113
	Understand the everyday routine	3.08	2.68	3.55	<0.001
	State one's birthday and age	2.00	1.63	2.44	<0.001
	Recall from short-term memory	1.88	1.67	2.10	<0.001
	State one's own name	1.03	0.50	2.11	0.934
	Know what the current season is	1.96	1.73	2.22	<0.001
	Know the environment	2.25	1.73	2.92	<0.001
	Make daily decisions	2.55	2.33	2.79	<0.001

A total of 20 items were entered simultaneously as the explanatory variables.

## Data Availability

To protect the participants' anonymity, the data will not be shared unless requested through an administrative procedure.

## References

[1] Institute of Population and Social Security Research (2012). *Population Projections for Japan (January 2012): 2011 to 2060*.

[2] Kshetrimayum N., Reddy C. V., Siddhana S. (2013). Oral health-related quality of life and nutritional status of institutionalized elderly population aged 60 years and above in Mysore City, India. *Gerodontology*.

[3] Saliba T. A., Ortega M. M., Goya K. K., Moimaz S. A. S., Garbin C. A. S. (2018). Influence of oral health on the quality of life of institutionalized and noninstitutionalized elderly people. *Dental Research Journal (Isfahan)*.

[4] Furuta M., Komiya-Nonaka M., Akifusa S. (2013). Interrelationship of oral health status, swallowing function, nutritional status, and cognitive ability with activities of daily living in Japanese elderly people receiving home care services due to physical disabilities. *Community Dentistry and Oral Epidemiology*.

[5] de Andrade F. B., Lebrão M. L., de Oliveira Duarte Y. A., Santos J. L. (2014). Oral health and changes in weight and waist circumference among community-dwelling older adults in Brazil. *Journal of the American Dental Association*.

[6] Scannapieco F. A., Shay K. (2014). Oral health disparities in older adults: oral bacteria, inflammation, and aspiration pneumonia. *Dental Clinics of North America*.

[7] Sarin J., Balasubramaniam R., Corcoran A. M., Laudenbach J. M., Stoopler E. T. (2008). Reducing the risk of aspiration pneumonia among elderly patients in long-term care facilities through oral health interventions. *Journal of the American Medical Directors Association*.

[8] de Andrade F. B. 1, Lebrão M. L., Santos J. L., Duarte Y. A. (2013). Relationship between oral health and frailty in community-dwelling elderly individuals in Brazil. *Journal of the American Geriatrics Society*.

[9] Ástvaldsdóttir Á., Boström A. M., Davidson T. (2018). Oral health and dental care of older persons-A systematic map of systematic reviews. *Gerontology*.

[10] Lee K. H., Plassman B. L., Pan W., Wu B. (2016). Mediation effect of oral hygiene on the relationship between cognitive function and oral health in older adults. *Journal of Gerontological Nursing*.

[11] Steinmassl P. A., Steinmassl O., Kraus G., Dumfahrt H., Grunert I. (2016). Is cognitive status related to oral hygiene level and appropriate for determining need for oral hygiene assistance?. *Journal of Periodontology*.

[12] Long-Term Care *Health and Welfare Services for the Elderly*.

[13] Japanese Ministry of Health, Labour and Welfare Long-Term Care Insurance Business Situation. https://www.e-stat.go.jp/stat-search/files?page=1&toukei=00450351&tstat=000001031648.

[14] Ethical Guidelines for Epidemiological Research (2013). *Ministry of Education, Culture, Sports, Science and Technology*.

[15] Ministry of Health, Labour and Welfare Japan (2015). *Guideline for Provision of Database for National Health Insurance Claim and the Specific Medical Checkup and Specific Health Guidance*.

[16] Ministry of Health, Labour and Welfare Japan (2017). *Security Guidelines for Health Information Systems*.

[17] Wu B. 1, Plassman B. L., Crout R. J., Liang J. (2008). Cognitive function and oral health among community-dwelling older adults. *Journals of Gerontology Series A: Biological Sciences and Medical Sciences*.

[18] Syrjälä A. M., Ylöstalo P., Ruoppi P. (2012). Dementia and oral health among subjects aged 75 years or older. *Gerodontology*.

[19] Sabbah W., Watt R. G., Sheiham A., Tsakos G. (2009). The role of cognitive ability in socio-economic inequalities in oral health. *Journal of Dental Research*.

[20] Noble J. M. 1, Borrell L. N., Papapanou P. N., Elkind M. S., Scarmeas N., Wright C. B. (2009). Periodontitis is associated with cognitive impairment among older adults: analysis of NHANES-III. *Journal of Neurology, Neurosurgery and Psychiatry*.

[21] Agüero-Torres H. l, Fratiglioni L., Guo Z., Viitanen M., von Strauss E., Winblad B. (1998). Dementia is the major cause of functional dependence in the elderly: 3-year follow-up data from a population-based study. *American Journal of Public Health*.

[22] Auyeung T. W., Kwok T., Lee J., Leung P. C., Leung J., Woo J. (2008). Functional decline in cognitive impairment--the relationship between physical and cognitive function. *Neuroepidemiology*.

[23] Putcha D., Tremont G. (2016). Predictors of independence in instrumental activities of daily living: amnestic versus nonamnestic MCI. *Journal of Clinical and Experimental Neuropsychology*.

[24] Sato E., Hirano H., Watanabe Y. (2014). Detecting signs of dysphagia in patients with Alzheimer’s disease with oral feeding in daily life. *Geriatrics and Gerontology International*.

[25] Shinagawa S., Adachi H., Toyota Y. (2009). Characteristics of eating and swallowing problems in patients who have dementia with Lewy bodies. *International Psychogeriatrics*.

[26] Dementia (2012). *A Public Health Priority*.

[27] UK to host G8 dementia summit, https://www.gov.uk/government/news/uk-to-host-g8-dementia-summit

[28] The Ministry of Health, Labour and Welfare Scientific Research Fund (2013). *Measures of the Dementia Prevalence and Life Function Disorders of Dementia in the City Areas, Dementia Countermeasure Total Research Project*.

